# Serological evidence of ebolavirus infection in bats, China

**DOI:** 10.1186/1743-422X-9-236

**Published:** 2012-10-13

**Authors:** Junfa Yuan, Yuji Zhang, Jialu Li, Yunzhi Zhang, Lin-Fa Wang, Zhengli Shi

**Affiliations:** 1 State Key Laboratory of Virology, Wuhan Institute of Virology-Chinese Academy of Sciences, Wuhan, Hubei, 430071, P R, China; 2 Huazhong Agriculture University, Wuhan, Hubei, 430070, P R, China; 3 Yunnan Institute of Endemic Diseases Control and Prevention, Dali, Yunnan, 671000, P R, China; 4 Commonwealth Scientific and Industrial Research Organization Livestock Industries, Geelong, Victoria, 3220, Australia

**Keywords:** Ebolavirus, Antibody detection, Bats

## Abstract

**Background:**

The genus *Ebolavirus* of the family Filoviridae currently consists of five species. All species, with the exception of *Reston ebolavirus*, have been found in Africa and caused severe human diseases. Bats have been implicated as reservoirs for ebolavirus. *Reston ebolavirus*, discovered in the Philippines, is the only ebolavirus species identified in Asia to date. Whether this virus is prevalent in China is unknown.

**Findings:**

In this study, we developed an enzyme linked immunosorbent assay (ELISA) for ebolavirus using the recombinant nucleocapsid protein and performed sero-surveillance for the virus among Chinese bat populations. Our results revealed the presence of antibodies to ebolavirus in 32 of 843 bat sera samples and 10 of 16 were further confirmed by western blot analysis.

**Conclusion:**

To our knowledge, this is the first report of any filovirus infection in China.

## Findings

Filoviruses are associated with acute fatal hemorrhagic diseases of humans and/or nonhuman primates when they spill over from their wildlife reservoir hosts. The family consists of two genera: *Marburgvirus* and *Ebolavirus*[1,2]. Five species of ebolavirus have been identified: *Ivory Coast ebolavirus*, *Sudan ebolavirus*, *Zaire ebolavirus* (EBOV), *Reston ebolavirus* (RESTV) and *Bundibugyo ebolavirus*. RESTV is the only known filovirus that does not cause severe disease in humans; however, it can be fatal in monkeys [3]. In 2009, infection of domestic pigs by RESTV was reported in the Philippines [4]. It was speculated that RESTV infected monkeys and pigs from an as yet unidentified host. Bats have been implicated as reservoirs for Marburgvirus [5] and Ebolavirus [6] in Africa and Asian country, the Philippines [7]. Previously, we have detected antibodies to the severe acute respiratory syndrome virus [8] and henipavirus [9] in bat sera in China. In this study, we conducted a surveillance study for the presence of ebolavirus in Chinese bat populations.

From 2006–2009, 843 bats were trapped within their natural habitat from several provinces in mainland China in accordance with animal ethics protocols approved by the Wuhan Institute of Virology, Chinese Academy of Sciences. Serum, pharyngeal and fecal swab samples were collected and stored as described previously [8]. Most animals were released back into their habitat. Those did not survive underwent necropsy.

**Figure 1:**
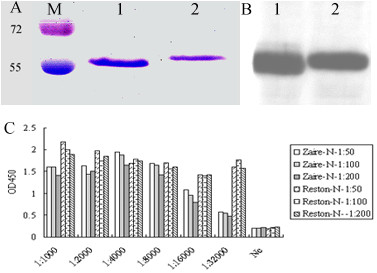
**Characterization of the truncated recombinant nucleocapsid protein of RESTV (Reston-NP) and EBOV (Zaire-NP).** Coomassie staining (**A**) and western blot analysis (**B**) of purified proteins separated by 10% SDS-PAGE. Lane M: molecular weight markers from Fermentas; Lane 1: Reston-NP; Lane 2: Zaire-NP. (**C**) ELISA analysis of Reston-NP and Zaire-NP using hyperimmune rabbit sera raised against the full-length Reston-NP.

The codon optimized nucleocapsid (NP) gene fragments (aa 410–653) of RESTV and EBOV were synthesized based on reference sequences downloaded from GenBank with reference numbers FJ621583 and L11365, respectively, and subcloned into pFastBac-HTb. The His-tagged truncated NP of RESTV (Reston-NP) or EBOV (Zaire-NP) were expressed in insect cells using the Bac-to-bac system (Invitrogen) and purified with a His.Bind Kit (Novagen) (Figure 1A & B) following the manufacturer’s instructions. Both the truncated Zaire-NP and Reston-NP strongly reacted to hyperimmune rabbit sera raised against the full-length NP protein of RESTV [10] by ELISA, although the optical density (OD_450_) readings to Reston-NP were higher than those to Zaire-NP under the same dilution (Figure 1C). The results indicated that both of these antigens were suitable for detection of ebolavirus antibodies. 

**Figure 2:**
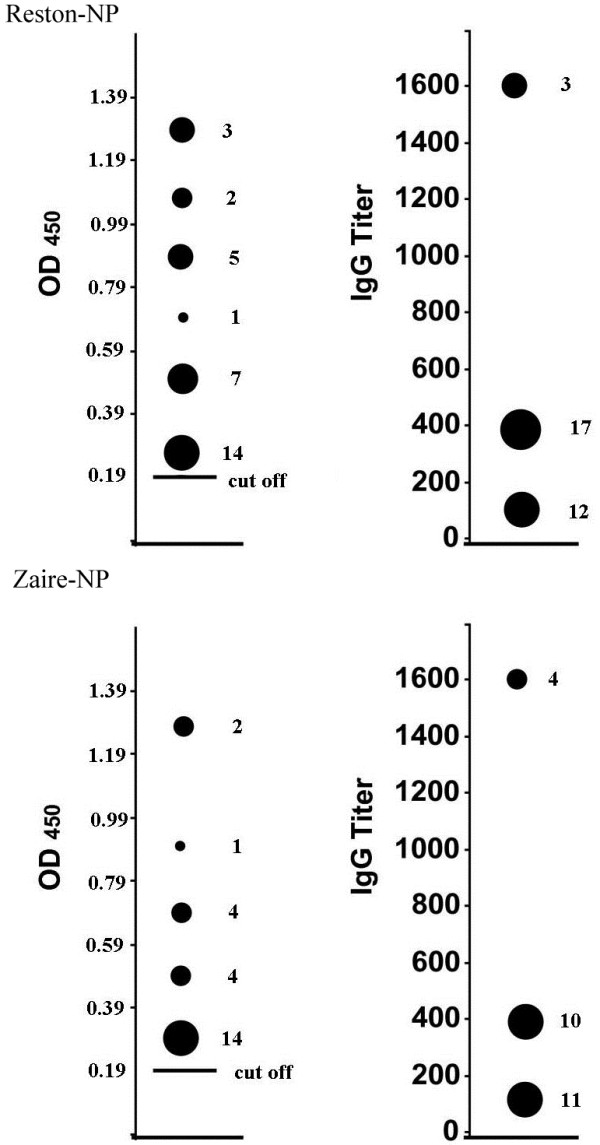
**Distribution of OD**_**450**_**readings and antibody titers among different positive bat sera.** The left panel shows the corrected OD_450_ readings at a serum dilution of 1:100. The right panel displays the antibody titers at an OD_450_ reading equal or greater than 0.19 (> 2-fold – ve sera). The number of sera at each OD_450_ or titer reading is shown to the right of the proportionally sized dots.

Considering the close geographical relationship of Chinese and Philippine bats, Reston-NP was used for the initial screening. ELISA plates were coated with the recombinant Reston-NP at approximately 100 ng/well and bat sera were tested in triplicates at a dilution of 1:100, followed by detection with horseradish peroxidase (HRP) conjugated Protein A/G (Pierce) at 1:20,000. Samples with a mean optical density 2.1-fold or higher than that of the negative control (OD_450_ value: 0.19) were considered positive. Positive serum samples were retested at dilutions of 1:100, 1:400, and 1:1600 against both Reston-NP and Zaire-NP (Figure 2).

**Figure 3:**
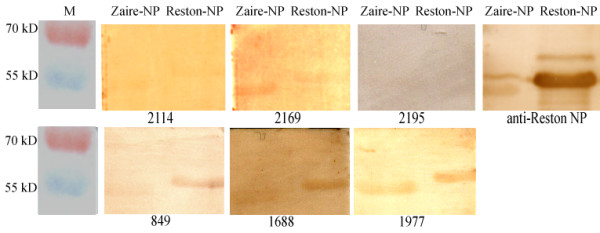
**Western-blot analysis of ELISA positive serum samples with recombinant Reston-NP and Zaire-NP expressed in*E. coli*.** The polyclonal antibody against the full-length nucleocapsid protein of RESTV was used as positive control. Sample no. 2195 was ELISA negative and used as negative control. The other 5 samples were western blot positive. Note: western blot no.1487, 1552, 1689, 1973 and 2166 is not presented due to the absence of signal in the scanned photograph.

A summary of the initial screening results is shown in Table 1. Of the 843 bat sera screened for antibodies to Reston-NP by ELISA, 32 were positive. These were from 10 of the 23 bat species collected from 5 different locations. Among the positive sera, 17 had OD_450_ readings higher than 0.19 at 1:400 and 3 at 1:1600 (Figure 2). These positive sera were further tested with the Zaire-NP protein and 25 were positive, and 10 samples had OD_450_ values higher than 0.19 at a 1:400 and 4 at a 1:1600. Sixteen bat sera with sufficient remaining quantity were further confirmed by western blot analysis with the recombinant NP expressed in *Escherichia coli*, and 10 were reactive to both the Reston- and Zaire-NP proteins (Figure 3, Table 2).

**Table 1:** Detection of antibody to RESTV nucleocapsid protein by ELISA

**Bat species**	**No. positive / No. tested (percent)**
**Megachiroptera**	
*Rousettus leschenaulti*	11/126(8.73%)
*Cynopterus sphinx*	2/2(100%)
**Microchiroptera**	
Hipposideridae	
*Hipposideros Pomona*	3/39(7.69%)
*Hipposideros spp.*	1/15(6.67%)
*Hipposideros cineraceus*	0/111
*Hipposideros armiger*	0/41
*Hipposideros larvatus*	0/21
*Rhinolophidae*	
*Rhinolophus affinis*	1/69(1.45%)
*Rhinolophus ferrumequinum*	0/15
*Rhinolophus sinics*	0/6
*Rhinolophus pusillus*	0/14
*Rhinolophus pearsonii*	0/3
*Rhinolophus spp.*	0/15
Vespertilionidae	
*Miniopterus schreibersii*	2/23(8.7%)
*Pipistrellus pipistrellus*	4/35(11.43%)
*Myotis ricketti*	4/83(4.82%)
*Myotis davidii*	0/5
*Myotis chinensis*	0/6
*Myotis daubentonii*	0/24
*Myotis fimbriatus*	0/2
*Myotis spp.*	3/118(2.54%)
*Scotophilus kuhli*	1/25(4%)
Unknown	0/2
Total	32/843(3.8%)

**Table 2:** Determination of ELISA titer and western blot reactivity against the truncated nucleocapsid proteins of RESTV (Reston-NP) and EBOV (Zaire-NP)

**Bat species and sample ID**	**Titer in ELISA**		**Western blot**	
	**Reston-NP**	**Zaire-NP**	**Reston-NP**	**Zaire-NP**
*Miniopterus schreibersii* (no:849)*	1600	1600	**+**	**+**
*Rousettus leschenaulti* (no:1263)	100	100	N	N
*Rousettus leschenaulti* (no:1276)	400	400	N	N
*Cynopterus sphinx (no:1487)*	400	400	**+**	**+**
*Hipposideros Pomona* (no:1552)	100	100	**+**	**+**
*Rousettus leschenaulti* (no:1688)*	100	100	**+**	**+**
*Rousettus leschenaulti* (no:1689)	400	N	**+**	**+**
*Pipistrellus pipistrellus* (no:1973)	400	400	**+**	**+**
*Pipistrellus pipistrellus* (no:1977)*	400	N	**+**	**+**
Myotis spp. (no:2114)*	400	400	**+**	**+**
*Rousettus leschenaulti* (no:2158)	400	100	N	N
*Rousettus leschenaulti* (no:2166)	100	N	**+**	**+**
*Rousettus leschenaulti* (no:2169)*	100	100	**+**	**+**
*Rousettus leschenaulti* (no:2183)	400	400	N	N
*Rousettus leschenaulti* (no:2190)	100	100	N	N
*Rousettus leschenaulti* (no:2211)	100	400	N	N

+: positive; N: no signal was detected; *: The results of western blot are shown in Figure 3.

A surrogate virus neutralization test was conducted using a recombinant env^-^ HIV-1 virus containing the luciferase reporter gene pseudotyped with spike glycoprotein proteins (GP) of EBOV (Zaire-GP) or RESTV (Reston-GP [11]). The plasmid encoding Zaire-GP (L11365) was kindly provided by Prof. Lijun Rong (University of Illinois at Chicago, USA). The Reston-GP gene was synthesized based on the RESTV genome sequence (FJ621583). Serum was serially diluted at 1:20 to 1:640 in 30 μl of medium and mixed with 30 μl of pseudovirus solution. The mixture was incubated for 1 h at 37°C and subsequently added, in triplicate, to 293T cells grown in a 96-well plate. The plate was incubated for 1 h at 37°C before being replenished with fresh medium and incubated for 48 h. Cells were lysed in 30 μl of lysis reagent (Progema) and luciferase activity was measured using a Luciferase assay kit (Promega). None of the positive bat serum inhibited entry of Reston-GP or Zaire-GP pseudotyped virus.

The Invitrogen OneStep RT-PCR Kit was used to screen ebolavirus RNA using the universal primers against the Filovirus L gene or N gene as described previously [12,13]. RNA was extracted using the QIAamp Viral RNA Mini Kit (Qiagen) following the manufacturer’s instructions. No filovirus-specific RNA was detected by one step RT-PCR among 143 tissue samples (spleen, liver or fecal swab) tested, therefore, virus isolation was not attempted.

In this paper, we presented serological evidence of ebolavirus infection in several bat populations in China. To our knowledge, this is the first report of any ebolavirus infection in this part of the world. The most significant prevalence of ebolavirus antibody was found among the *Rousettus leschenaulti, Pipistrellus pipistrellus and Myotis* species. Several serum samples have relatively high titer to both Reston-and Zaire-NP.

There are several possibilities to account for the failure in detecting neutralizing antibodies. In general, bats seem to produce lower level of neutralizing antibodies in response to viral infection, possibly due to the lower affinity of the bat antibodies [14]. Alternatively, it is possible that one or more as-yet-unknown ebolaviruses are circulating among the bat populations sampled in this study, producing antibodies cross-reactive with, but not neutralizing EBOV or RESTV. Also, a new ebolavirus species closely-related with EBOV but not the RESTV might be missed during the initial screening using Reston-NP solely. An initial screening for eboalvirus antibodies should be conducted by using ELISA with a 1:1 mixture of recombinant NP of EBOV and RESTV to detect wider range of ebolavirus species. Recently, a genetically distinct filovirus was found in dead insectivorous bats in Spain [15], suggesting that filoviruses have a wider host range and geographical location than previously thought. The unsuccessful identification of ebolavirus-related genes in the samples is likely attributable to the often low-level of virus replication, the similarly transient nature of the infection in bats or the sequence mis-match of the PCR primers used and the target sequence of the potential unknown ebolavirus genomes.

There are approximately 120 species of bats distributed throughout China. Bat species in the genera *Rousettus*, *Hipposideros, Myotis*, *Miniopterus* and *Pipistrellus* naturally reside in trees, buildings and caves that can be in close proximity to human residential areas, increasing the potential of zoonotic transmission from bats to humans. There is evidence of human infection of ebolavirus in central Africa as a result of direct bat-to-human transmission [16]. In the Guangdong and Hainan provinces of China, local populations customarily eat large bats, such as Leschenault's rousette. The preliminary results presented in this study highlight the need to continue and expand surveillance for ebolavirus or related viruses in China and elsewhere in the Asian continent.

## Competing interests

The authors declare that they have no competing interests.

## Authors’ contributions

JY and YZ independently performed protein expression and ELISA. JL performed the RNA extraction and RT-PCR. YZ provided samples and information about bats. LFW and ZS conceived the project and provided overall scientific oversight. All authors contributed to the preparation of the final manuscript. All authors read and approved the final manuscript.
